# The Relationship Between Parental Play Beliefs, Preschoolers’ Home Experience, and Executive Functions: An Exploratory Study in Ethiopia

**DOI:** 10.3389/fpsyg.2020.00624

**Published:** 2020-04-17

**Authors:** Biruk K. Metaferia, Zsofia K. Takacs, Judit Futo

**Affiliations:** ^1^Doctoral School of Psychology, ELTE Eötvös Loránd University, Budapest, Hungary; ^2^Institute of Education, ELTE Eötvös Loránd University, Budapest, Hungary; ^3^Institute of Psychology, ELTE Eötvös Loránd University, Budapest, Hungary

**Keywords:** executive function, parental belief, home activity, preschool, play belief

## Abstract

Although research has highlighted the importance of home experience and especially of play in early brain development, the value of this factor for executive function (EF) development has not received the attention it deserves. The purpose of the present study was to investigate the link between parental play beliefs and preschoolers’ play frequency at home on the one hand and their EF skills on the other. Additionally, other types of home activities were also assessed. A total of 102 preschoolers (45 girls; mean age = 62.08 months; *SD* = 7.66 months; range, 50–74 months) with their parents (mean age = 35.21 years; *SD* = 6.96 years) representing low to middle socioeconomic status (SES) families in Ethiopia participated in the study. Results revealed that children’s home activities (frequency of breakfast at home, spending mealtime together with family, participation in peer play, participation in pretend play, and participation in arts and crafts) and parental play support were significantly positively correlated with their performance on EF tasks. Hierarchical regression analyses controlling for age and SES showed that parental play support and frequency of breakfast at home were medium-sized predictors (β = 0.36, *p* < 0.001 and β = 0.31, *p* = 0.001, respectively) explaining a significant level of variance in inhibitory control, while participation in arts and crafts at home was a significant predictor (β = 0.22, *p* = 0.03) of children’s performance on a visual–spatial working memory (VSWM) task. In conclusion, parental play support and preschoolers’ home activities are important factors linked with EF development in early childhood.

## Introduction

Early childhood is an important period of life when marked developmental improvement of executive function (EF) occurs (Carlson, 2005; Fay-Stammbach et al., 2014). Inadequate attainment of EF skills in this period of life has been associated with a number of problems such as developmental psychopathology (Pennington and Ozonoff, 1996), physical aggression (Séguin and Zelazo, 2005), and problems related to school readiness and academic success (Blair, 2002; Diamond, 2007a). Home experience (e.g., parent–child interaction and activities, parental scaffolding, and attachment security) is proposed to be among the important determinants influencing early brain development (Carlson, 2009).

EF denotes a set of interconnected cognitive skills that guide and support self-regulation of thought, action, and emotion (Anderson, 2002; Séguin and Zelazo, 2005). There is no general consensus on whether children’s EF can be separated into components and, if so, how many of them (Lehto et al., 2003; St Clair-Thompson and Gathercole, 2006; Wiebe et al., 2008; Diamond, 2013a). According to Miyake et al. (2000), it encompasses three basic neurocognitive abilities in adulthood: the ability to withhold irrelevant and unnecessary thoughts and behaviors that obstruct accomplishing a given task (inhibitory control; McClelland et al., 2007b); the ability to hold information in mind (working memory; Blakey and Carroll, 2015); and the ability to flexibly adjust one’s thinking and behavior as per the demands of a given situation (cognitive flexibility; Davidson et al., 2006).

Inhibitory control enables children to regulate their own attention, thoughts, emotion, and behavior from their internal tendency and external temptation (Diamond, 2013a). An example for the role of this skill in a playground could be that children need inhibitory control in order to be able to wait for their turn and stick to the rules of games. In the classroom context, children with stronger inhibitory control have a better control over their behavior even in the presence of distractors. For instance, children with well-developed inhibitory control skills could inhibit their impulsive behavior, obey classroom rules and regulations, keep on focusing their attention, and do a class activity while resisting distraction (McClelland et al., 2007a).

Working memory (WM) denotes the mental process that enables us to maintain information in a readily accessible state to use it (Cowan et al., 1999; Diamond and Ling, 2016). It serves as a form of mental workspace for most cognitive processes (Fukuda et al., 2010). It is vital for reasoning and problem solving, as these mental processes require holding information in mind and analyzing and synthesizing them for a given purpose (Diamond and Ling, 2016).

Cognitive flexibility is the ability to switch between competing response alternatives as task demands change (Miyake et al., 2000; Davidson et al., 2006). For instance, if a given problem is not solved using one technique, then one needs cognitive flexibility to think of another technique to approach it with. Such flexibility skills are very important especially in a novel situation (Diamond and Ling, 2016). The result of extended investigation on the operation of cognitive flexibility in childhood indicated that it involves the use of a combination of other cognitive skills together so that the new response set is activated; the response set that was activated before is inhibited, and the response rules are retained and processed in working memory (Davidson et al., 2006; Chevalier and Blaye, 2008). According to Buss and Spencer (2014), flexibility in the Dimensional Card Sorting Task can be attained by modulating specific features of neural connectivity within and between the dimensional attentional system and the cortical fields sensitive to different features of the stimulus.

### Home Experience and Early Executive Function Development

The development of EF is determined by the interplay between environmental and genetic factors (Posner and Rothbart, 2000; Kochanska et al., 2009). Factors such as socioeconomic status (Noble et al., 2005; Sarsour et al., 2011), parent–child interaction (Bernier et al., 2010, 2012; Rhoades et al., 2011; Blair et al., 2014), and parental scaffolding (Hughes and Ensor, 2009; Bernier et al., 2010; Hammond et al., 2012) are among the most important home experience variables associated with developmental differences in EFs in early childhood. On the other hand, severe problems associated with child’s experiences at home (e.g., abuse, maltreatment) are connected with deficits in early EF development (Pechtel and Pizzagalli, 2011).

For most children, the home creates the environmental context in which they could learn socialization (Dreyer and Dreyer, 1973). In this regard, the time for the family to be together such as mealtime could create opportunities for a myriad of learning to take place on the part of children (Dreyer and Dreyer, 1973). In family mealtime, parents create the setting in which the meal is conducted and bring to the table expectations for behavior (Fiese and Schwartz, 2008). Beyond learning specific behaviors centered about dinner, children also learn the general roles, rules, and values of family living coalesce in this setting (Dreyer and Dreyer, 1973). Moreover, Snow and Beals (2006) and Weizman and Snow (2001) reported improved language development and academic achievement in children when mealtimes are characterized by responsiveness to children’s questions and when behavior is well regulated. Furthermore, there are also evidence indicating that frequency of family mealtimes and family climate during shared mealtimes are related to the behavior and development of a child. For instance, children with families spending mealtime together have shown fewer behavior problems (Hofferth and Sandberg, 2001) and vocabulary growth (Snow and Beals, 2006).

Breakfast is another important element of children’s everyday lives at home that could affect their cognitive function. The importance of breakfast consumption is usually stressed by parents and educators assuming that it is important for children’s successful learning (Pollitt and Mathews, 1998). In fact, the findings of studies investigating the associations between breakfast consumption and cognitive function depict several inconsistencies that could be attributed to differences in study designs and study population (Micha et al., 2010; Widenhorn-mu et al., 2020). In their review study, Adolphus et al. (2016) explicitly stated that very few studies investigating the breakfast–cognition link deal with the nutritional value, which seems to be a general limitation of the literature examining the issue. Most studies, instead, used other means of measures such as frequency of breakfast, comparing breakfast with no breakfast, and blood glucose monitoring (see Adolphus et al., 2016). However, the overall findings of the studies have indicated that breakfast can positively impact cognitive functioning (Cooper et al., 2011). There might be several possible explanations for this finding. The first would be that having breakfast regularly could enhance the nutritional value that the child receives, which might have a direct positive impact on brain development (Datar and Nicosia, 2012). The second explanation would be that having or not having breakfast on the morning of the cognitive function tests could have a temporary effect on children’s performance on the tasks (see Datar and Nicosia, 2012). As children tend to sleep for a longer time during the night and their energy reserve is smaller, by the morning, it is more likely that their stored glycogen is depleted (Datar and Nicosia, 2012). This results in their brain experiencing a shortage of energy in the morning that could affect children’s cognitive functions (Wesnes et al., 2003; Datar and Nicosia, 2012).

Physical activity is another important home experience that has implications for cognitive and social development (Diamond, 2007b). There is evidence from experimental studies with children indicating that cognitively engaging exercises (rule-based ball games, for example) have a stronger effect on the brain than cognitively non-engaging exercises (like running) (see Sibley and Etnier, 2003; Tomporowski et al., 2008). There has been a relatively well-established association between motor skills and EF in children (Fels et al., 2014; Maurer and Roebers, 2019). The results of an investigation by Campbell et al. (2002) examining the role of motor activity in preschoolers’ behavioral control indicated that higher levels of physical activity predicted higher inhibition performance. Other studies focusing on the effects of physical activity on different aspects of cognitive functions show that physical activity can improve attention (Chaddock et al., 2010; Davis et al., 2011) and working memory (Mcmorris et al., 2011; Niederer et al., 2011). Another evidence that could demonstrate the association between the two is that, most of the time, motor and cognitive problems occur together in children with neurodevelopmental disorders (Diamond, 2000; Hellendoorn et al., 2015).

Motor coordination, which is the aspect of motor skills, is strongly associated with EF in both typically developing children (Fels et al., 2014) and children with developmental coordination disorder (Wilson et al., 2012; Leonard, 2016). Studies investigating the association of both fine and gross motor coordination with EF in children and adolescents (Livesey et al., 2006; Rigoli et al., 2012; Luz et al., 2014) depicted that children and adolescents with higher levels of fine/gross motor coordination outperform their counterparts in cognitive performance activities. Considering the mechanisms of physical activities in childhood, Best (2010) argued that much of the physical activity and exercise comes through children’s involvement in group activities that demand complex cognition to cooperate with teammates, forecast teammates’ and opponents’ behaviors, plan and employ strategies, and flexibly adapt oneself to dynamic task demands (Best, 2010), almost all of which are similar to what EF tasks demand in children’s executive processes (Banich, 2009).

Social interactions are also among the important factors that might influence the development of EFs (Moriguchi’s, 2014). Diamond (2013b) argues that activities that make children happy and proud such as music making, singing, dancing, and sports help to improve their EF. Such kind of activities “challenges EF by requiring focused attention over sustained periods, holding complex sequences in mind, and the self-control needed to put in the hours of practice when there are temptations to do other things and when one may be frustrated with one’s progress at times” (Diamond, 2013b, p. 216).

#### Play and Executive Function in Early Childhood

The importance and contribution of play to the physical, cognitive, social, and emotional developments of children is grounded in a strong body of research (e.g., Pellegrini and Smith, 1998; Burdette and Whitaker, 2005; Ginsburg, 2007). Bierman and Torres (2016) illustrated how play helps children’s EF development: through taking turns and thinking before acting, children practice and learn self-control skills; through memorizing the rules of a game, they improve their working memory; and through role playing and taking the perspective of other children in the group, they refine cognitive flexibility skill. There are investigations (e.g., Berk and Meyers, 2013; Pierucci et al., 2014; Thibodeau et al., 2016) indicating a positive association between children’s spontaneous pretending and EF skills. In the current literature, however, it seems that play type/form other than pretend play (e.g., peer play, solitary play, motor play) get little attention especially in relation to the development of EF in early childhood. Besides, there are other home experience factors such as motor (gross and fine) activities and sport and physical activities that could have valuable contribution to physical, cognitive, emotional, and social development (see Pellegrini and Smith, 1998). However, we know little about the link between these and other home experiences (such as frequency of breakfast at home, spending mealtime with family, academic-related activities at home) and EF.

In connection to children’s play, parents hold different views, which range from perceiving play as primarily entertainment all the way to play as a means for a range of developmental benefits to children (Farver and Howes, 1993; Fisher et al., 2008). In this regard, Fogle and Mendez (2006) developed a scale to measure parents play beliefs using low-income Africa-American mothers in the United States and came up with two views of play (play support and academic focused). The play support belief represented a view that play is a means for important developmental outcomes beyond being entertaining activities for children. The academic focused belief, on the other hand, represented a view that play is a less worthwhile activity for children’s development in contrast with explicit academic activities, such as reading to a child. Such beliefs of parents could impact the extent to which they support play activities at home, the nature of play activities, and the degree of their involvement in their children’s play (Farver and Wimbarti, 1995; LaForett and Mendez, 2017). In this regard, a comparison of parents’ involvement in their children’s play indicated that mothers, who valued play for its educational and cognitive benefits, were more likely to join their children’s play, while mothers who hold a belief that the value of play is merely for entertainment did not exert meaningful efforts to facilitate their children’s play (Farver and Howes, 1993; Farver and Wimbarti, 1995). There is a growing body of literature underlining the important contribution of parents’ involvement in their children’s play in improving their developmental outcomes (Md-Yunus, 2007; Lin and Yawkey, 2013).

### Sociocultural Context in Ethiopia

Ethiopia is a multiethnic and ecologically diverse country with a population of around 109 million (World Bank, 2019b). According to the World Bank report of 2018, Ethiopia’s gross domestic product (GDP) was estimated as 84.36 billion USD (World Bank, 2019b). Only 20% of the population are living in urban areas (Wold Bank, 2019a).

Even if Ethiopia is home for a number of languages, most of the preschoolers are monolingual. Seventy-one percent of them live with both parents (Better Care Network, 2015) who are important figures in molding their behavior and thinking patterns. Children at the age of 4 go for a 3-year preschool program (Admas, 2016) where great emphasis is put on literacy and far less on play (Tigistu, 2013). Academic-oriented content, rote memory, and drill are the most commonly practiced teaching methods in preschools (Admas, 2016). Parallel to this phenomenon, parents of preschoolers expect their children to read, write, and practice arithmetic (Admas, 2016). These parental demands could be the basic reasons driving preschools to focus on academics at the expense of other important aspects of development (Tigistu, 2013).

As kindergarten/preschool is an urban phenomenon in the country (MoE, 2016), other modalities of pre-primary education (namely, “0” class, and Child-to-Child programs) are delivered in the rural part of the country in order to improve children’s enrollment in pre-primary education. In the Child-to-Child program, older children support younger ones in their neighborhood (MoE, 2016). On the other hand, “0” class program is launched in the primary schools where young children get prepared for the next level of schooling.

### Present Study

While many investigations have proposed that the type and nature of children’s home experiences (Noble et al., 2005; Bernier et al., 2012; Hammond et al., 2012) play an important role in their school readiness, cognitive development, and self-regulatory skills, only a handful of studies have directly investigated how these experiences (children’s home activities and their parents’ play beliefs) are related to EF development. Additionally, the majority of this handful of studies was conducted with western samples and under the western sociocultural context. Moreover, to the best of our knowledge, the current investigation is the first of its kind to study the link between parents’ play beliefs and their preschoolers’ EF skills. Furthermore, as far as our knowledge goes, there is no study investigating the role of children’s everyday activities at home that includes both play and other experiences such as frequently of breakfast, mealtime together with family, sports, and their roles in EF skills. Including all these components of home activities into our study helped us to provide a more comprehensive view on the connection between children’s home environment and the development of their EF skills including the exploration of the relative importance of children’s different home experiences in relation to their EF skills. Therefore, exploring possible associations between preschoolers’ home experiences (parents’ play beliefs and preschoolers’ home activities) and EF development would fill a gap in the current literature.

Taking the important role of children’s home experience for development into consideration, the purpose of the current study was to investigate the link between Ethiopian preschoolers’ activity at home and parental play beliefs and development of their EFs. To achieve this purpose of the study, the following research questions were formulated:

(1) Is the frequency with which preschoolers engage in different forms of play at home associated with their EF skills?
(2) Do parental play beliefs significantly relate to children’s EF skills?
(3) What is the relationship between preschoolers’ home activities (such as spending mealtime with family, breakfast experience at home, academics-related activities, and sports and physical activities) and their EF skills?

Regarding the first research question, we hypothesized that the more children spend playing (especially pretend play and peer play) at home, the better their performance in EF tasks (Kelly and Hammond, 2011; Bierman and Torres, 2016). On a related note, we hypothesized that children with parents who believe that play has an important role in children’s development would have better performance in EF tasks. On the other hand, we did not have specific hypotheses regarding the relationship between different home activities (such as academics-related activities, spending mealtime with family, breakfast experience at home, and sports and physical activities) and EF; this part of the study was exploratory. It should be noted that we originally intended to test our research questions on all three EF components (working memory, inhibitory control, and cognitive flexibility), however, we could not use the data of the switching task intended to measure cognitive flexibility for statistical analyses due to its low level of variance (see more details under *Measures*).

## Methods

### Participants

Families were recruited from a variety of preschools in Ethiopia, which ranged from small to large city area preschools representing lower to middle-class areas. One hundred two preschoolers (57 boys and 45 girls; mean age = 62.08 months; *SD* = 7.66 months; age range, 50–74 months) with their parents (34 men and 68 women; mean age = 35.21 years; *SD* = 6.96 years; and age range, 25–70 years) participated in the present study. The person who filled in the questionnaire was the mother in 55% of the cases, the father in 32%, the grandmother or the grandfather of the child in 2%, or some other relatives living with the child in 11%. The participating children in the study were preschoolers who (at the time of the data collection) have been attending preschool for less than a year (24.5%), between a year and 2 (51.0%), and 3 years (24.5%). The demographic characteristics of the participants are presented in Table 1.

**TABLE 1:** Sample demographics.

**Variables**	**Frequency**	**Percent**
**MOTHER’S EDUCATION LEVEL**		
Elementary school complete	16	15.7
High school complete	42	40.8
College diploma	30	29.1
University degree	11	10.7
Graduate degree (master’s or above)	3	2.9
Not reported	1	1
**FATHER’S EDUCATION LEVEL**		
Elementary school complete	10	9.8
High school complete	24	23.5
College diploma	27	26.5
University degree	21	20.4
Graduate degree (master’s or above)	15	14.6
Not reported	5	4.9
**GROSS HOUSEHOLD ANNUAL INCOME IN BIRR**		
0–24,999	8	7.8
25,000–49,999	16	15.7
50,000–79,999	25	24.5
80,000–99,999	17	16.7
100,000–149,999	20	19.6
150,000–199,999	12	11.8
200,000 or more	2	2.0
Not reported	2	2.0

#### Procedures

Participant preschool centers were selected from three cities in Ethiopia (Addis Ababa, Ambo, and Hawassa) using convenience sampling. The directors of the centers gave written consent to participate in the study, after which parents were sent a consent form (about their own and their children’s participation). To the parents who gave consent, a questionnaire was sent. Children of these parents were taken out of the classroom in the preschool for an individual testing session (about 20–25 min) including neuropsychological tests of executive functions. The order of the tasks was the same for all participant: children started with a go/no–go task followed by a switching task, and they all finished with a test of VSWM task. One-on-one testing was conducted in a separate, quite room. The procedure implemented in the current study was approved by the Eötvös Loránd University’s Research Ethics Committee (issue number: 2017/209). The data collection period ranges from July to September of 2017.

### Measures

Children’s executive function skills were assessed by neuropsychological tests applied in a one-on-one testing session in the preschool. A questionnaire was sent to the parents in order to collect data about preschoolers’ home activities and their parents’ play beliefs. The questionnaire used consisted of three parts: a scale including demographic questions, a scale regarding the frequency of different home activities (developed by the investigators of the present study), and a scale concerning the parents’ beliefs about play (PPBS).

#### Demographic Information

Demographic data included the respondent’s gender and relationship with the child, the child’s gender, age, the education levels of the father and the mother, the family gross annual income, and the child’s years of enrollment in a preschool program. The education level of the parents was rated on a 5-point scale ranging from 1 (Elementary school complete) to 5 (having Graduate Degree/masters or above). Gross household annual income was rated on 7-point scale ranging from 1 (0–24,999 Birr) to 7 (200,000 Birr or more). A socioeconomic status (SES) variable was constructed by averaging the average of parents’ education and their annual gross income.

#### Parent Play Beliefs Scale

The final version of Fogle and Mendez’s (2006) Parent Play Beliefs Scale (PPBS) was used to measure parents’ perspectives on the role of play in their children’s development. The scale was developed using African-American mothers with their preschool-age children in Head Start. The scale consists of 25 items rated on a 5-point Likert-type scale rated from 1 (disagree) to 5 (very much agree). In Fogle and Mendez’s (2006) investigation, the principal component analysis yielded two subscales: play support and academic focus. The play support subscale consisted of 17 items (e.g., “through play my child develops new skills and abilities,” and “I can help my child learn to control his or her emotions during play”) reflecting a view that play has the potential to offer a range of developmental benefits to children on top of being an entertaining activity. Academic focus, on the other hand, consisted of eight items (e.g., “I do not think my child learns important skills by playing,” and “I would rather read to my child than play together”) reflecting views that academically oriented activity rather than play is important for their children’s development.

In their validation study, Fogle and Mendez (2006) reported that the internal consistency of the two subscales was high (α = 0.90 and 0.73, respectively). In the current study, the same subscales were used, and they again showed good internal consistency (α = 0.88 and 0.75, respectively) after deleting an item from the academic focused subscale. The bivariate correlation of the two subscales depicted a significant negative correlation (*r* = −0.21, *p* = 0.043).

#### Home Activities Scale

The home activities scale was developed by the investigators of the present study to assess children’s frequency of different activities in their home. It consisted of 10 items corresponding to 10 activities that we aimed to assess: academics-related activities, spending mealtime with family, breakfast at home, frequency of pretend play, motor play, fine motor activities, arts and crafts, solitary play, peer play, and sports and physical activity (see Supplementary Appendix for the measure). In the scale, parents were presented with a list of preschoolers’ daily routines after preschool and asked how often their children engage in the activities (e.g., how often do their children engage in arts and craft, academic activities, pretend play, peer play, or solitary play). The participants used a 5-point response Likert scale, ranging from “very rarely/less than once a week” (1) to “very frequently/most of the time during the day” (5), to indicate the extent to which their children were involved in the activities included in the scale. The validity of the home activities scale was assured in two ways. First, the principal investigator is familiar with the family routines and childcare practices in Ethiopia. Second, feedback was collected from experts familiar with the situation on the validity of the scale during its development.

#### Translation of the Survey

The questionnaire was translated to the target language (Amharic). The process involved three independent translators: two educational psychologists with a master’s degree and one applied developmental psychologist with a Ph.D. level of education. The translators were native speakers of Amharic but were fluent English speakers. The first author collected the three translations, checked the discrepancies, and made a first version. Finally, the Amharic questionnaire was back translated to English by an English language expert with Amharic mother tongue to compare the equivalence of the original scales in English and the target scale in Amharic. Based on the back translation, important revisions were made.

#### Inhibition Task

The fish–shark go/no–go task (Wiebe et al., 2012), where children were instructed to respond to the picture of a fish (go stimulus) by a button press, but not for the picture of a shark (no–go stimulus), was used on a laptop computer using PsychoPy 1.85.1 version (Psychological Software Tools; Peirce, 2008) in order to assess children’s inhibitory control skills. On each trial, the stimulus was presented for 1,500 ms if the participant did not respond earlier (see Wiebe et al., 2012). There were six practice trials (four go and two no–go trials). When the participant demonstrated that they understood the instructions, the test was started. The test consisted of 60 trials, two-thirds of which were go trials and one-third were no–go trials. Sensitivity (*d*′) was computed using signal detection theory that indicates the extent to which one responds differentially to two groups of stimuli (Macmillan and Creelman, 2004). Hits (correct go trials) and false alarms (incorrect no–go trials) were used in computing sensitivity. A higher value of sensitivity indicates better performance in the task (Macmillan and Creelman, 2004).

#### Switching Task

Another version of the go/no–go task was applied in order to assess children’s cognitive flexibility: using different stimuli (a picture of a cat and a tiger) and four blocks in which the rule was switched. Accordingly, in the first and third blocks, participants were instructed to make a button response whenever the picture of a cat (go stimulus) appeared at the center of a laptop computer screen and refrain from responding in case of the picture of a tiger (no–go stimulus). In the second and fourth blocks, the participants completed tasks with the reversed stimulus–response rules as in the first and third blocks. Under each block, 16 go and 8 no–go trials were used. For each trial, just like in the fish–shark go/no–go task, the stimulus (cat or tiger) appeared on the screen for 1,500 ms if the participant did not press the button earlier. Before starting the first block, there were six practice trials including four go trials and two no–go trials. In addition, participants were informed about the corresponding rule of every block before the beginning of each block of the task. Sensitivity was calculated for each block the same way as in the go/no–go task. Following this, average sensitivity scores of switching blocks were calculated by taking the average of the sensitivity difference of switching blocks (i.e., difference in sensitivity of the third and the first blocks and the difference in sensitivity of the fourth and the second blocks), with higher scores indicating better cognitive flexibility skill. However, following this calculation, the very small range of average sensitivity scores [−1.56, 0.88], where the possible range varies between −6.081 and 6.081, became apparent. Because of the observed lack of discriminative power, the scores of the switching task were excluded from the analysis.

#### Visual–Spatial Working Memory Task

A visual–spatial working memory (VSWM) test, the “Mr. Peanut” task (Kemps et al., 2000), was chosen to be used in the current study. We used a computerized version that was based on the work of Morra (1994). In the task, preschoolers are presented with a clown figure called “Mr. Peanut” on a laptop computer screen where he decorates himself by putting stickers at any of the 14 different locations on his body. After that, he disappears from the screen and reappears without the stickers. Children are requested to locate the part of Mr. Peanut’s body that he decorated with stickers before they were removed (for more details, see Morra, 1994). The test is carried out after a practice session where the child completed three practice trials. The test starts only when the participant correctly responds for all the three practice trials. The program offers the opportunity for the participant to change their response by deleting the sticker they already located.

In the test phase of the task, participants were presented three trials one after the other on each level (starting with one sticker). When they successfully passed a given level by correctly responding to at least one of the three trials, they were moved up to the next level (at each consecutive level, the number of stickers increases by one). The test automatically stops if a participant makes three consecutive mistakes on any of the given levels. When calculating the scores, the rule outlined by Morra (1994) was applied: in case the participant correctly responded to at least two of the three trials on a level, he/she is scored 1 point, and for correctly responding for only one trial on a level recorded 1/3 point. The total score is the sum of the scores that participants obtained on each of the levels they successfully passed through.

#### Data Analysis

SPSS v25.0 for Windows (IBM Corporation, Armonk, NY, United States) was used for performing the analyses. Out of the 131 questionnaires that were sent out, 116 (89%) were returned, and six of these were excluded from analysis as they had a lot of missing values. We could not test eight children with the EF tasks due to their absence from preschool during data collection. In addition, as 17 participants experienced a technical problem in the fourth block of the switching task, they were excluded from the analysis of switching variable. In addition, seven children with missing values on the working memory test and six with negative sensitivity (*d*′) score on the go/no–go task were excluded from analysis of working memory and inhibitory control variables, respectively. Negative *d*′ score happens when hit rate is lower than false alarm rate that can occur as a result of response confusion (Stanislaw and Todorov, 1999). However, one child with negative *d*′ score in the fourth block of switching task was not excluded from the analysis, as it could be an indicator of perseverance. Moreover, the assumption of normality and homogeneity of variance in addition to outlying scores were inspected in all the analyses. Two outliers found in parental play support scores and one from switching scores were excluded from the analyses.

Bivariate correlation was computed to evaluate the relationship between preschoolers’ performance in EF tasks on the one hand and their home activities and parents’ play beliefs on the other. Analysis of the home activities was run using individual items. Furthermore, we conducted hierarchical regression analyses predicting children’s performance on the EF tests from significant correlates from the home activities and the parental play beliefs variables controlling for children’s age and SES. The control variables of age and SES were entered first in the regression models, followed by entering predictors that showed significant associations with the dependent variable based on the results of correlation analyses. In analyzing inhibitory control skills, we used *d*′, which is calculated using hit (correct go trial) and false alarm (incorrect no–go trials) rates.

## Results

### Descriptive Statistics

Table 2 presents descriptive statistics for preschoolers’ executive functions, home activities, and parental play belief scores. It is presented in the way that data were used in the analysis, i.e., the excluded outliers from the analysis were also excluded from the descriptive statistics.

**TABLE 2:** Mean, standard deviation, and minimum and maximum scores for children’s EF performance, home activities, and parental play beliefs.

**Variables**	***N***	**Mean**	***SD***	**Min–Max**
SES	95	3.27	1.09	1.50–6.25
**CHILD’S EF**				
VSWM	95	2.50	0.80	0.67–4.00
Inhibitory control	95	2.57	1.00	0.25–4.20
Cognitive flexibility	84	–0.33	0.59	−1.56–0.88
**CHILD’S HOME ACTIVITY**				
Academic skills practice after preschool	102	3.12	1.15	1–5
Mealtime together with family	99	3.60	1.02	2–5
Breakfast at home	99	2.88	1.09	1–5
Engage in pretend play	99	3.10	1.27	1–5
Engage in motor play	102	3.27	1.11	1–5
Engage in fine-motor activities	100	3.25	1.18	1–5
Participate in arts and crafts	95	2.43	1.40	1–5
Engage in solitary play	97	2.30	1.45	1–5
Play with peers	101	3.29	1.30	1–5
Do sports and physical activities	101	2.78	1.18	1–5
**PARENTS’ PLAY BELIEFS**				
Parental belief: academic focused	95	18.80	4.67	10.00–29.00
Parental belief: play support	92	72.19	6.80	55.00–85.00

### Correlational Analyses

In bivariate correlation analyses, we examined whether the sociodemographic variables (children’s age and SES), child’s home activities, and parents’ play beliefs variables were correlated with children’s performance in EF tasks, as shown in Table 3. Both age and SES demonstrated significant, positive correlations with performance on the go/no–go task and on the VSWM test. Spending mealtime together with the family, having breakfast at home, frequency of pretend play, and peer play showed significant positive relations with children’s performance on the go/no–go task. Moreover, parental play support demonstrated a significant positive correlation with performance on the go/no–go task. Frequency of having breakfast at home and the frequency of participation in arts and crafts activities also showed significant positive correlations with preschoolers’ performance on the Mr. Peanut VSWM task.

**TABLE 3:** Bivariate correlations between study variables.

	**1**	**2**	**3**	**4**	**5**	**6**	**7**	**8**	**9**	**10**	**11**	**12**	**13**	**14**	**15**	**16**
1. Child’s age	1															
2. SES	0.06 *N* = 95	1														
3. Visual–spatial working memory	0.41** *N* = 94	0.21* *N* = 88	1													
4. Inhibitory control	0.38** *N* = 94	0.46** *N* = 88	0.41** *N* = 89	1												
5. Academics-related activities	0.19 *N* = 101	−0.05 *N* = 95	0.06 *N* = 95	0.01 *N* = 95	1											
6. Mealtime with family	0.07 *N* = 98	0.32** *N* = 92	0.01 *N* = 93	0.25* *N* = 92	−0.03 *N* = 99	1										
7. Breakfast at home	0.21* *N* = 98	0.23* *N* = 93	0.21* *N* = 92	0.34** *N* = 92	0.25* *N* = 99	0.04 *N* = 96	1									
8. Pretend play	0.33* *N* = 98	0.27* *N* = 93	0.14 *N* = 92	0.39** *N* = 92	0.05 *N* = 99	0.28** *N* = 97	0.34** *N* = 97	1								
9. Motor play	0.15 *N* = 101	0.11 *N* = 95	0.02 *N* = 95	0.19 *N* = 95	0.04 *N* = 102	0.00 *N* = 99	0.16 *N* = 99	0.18 *N* = 99	1							
10. Fine motor activities	0.15 *N* = 99	0.10 *N* = 93	0.20 *N* = 93	0.20 *N* = 93	−0.05 *N* = 100	0.17 *N* = 98	0.05 *N* = 98	0.18 *N* = 98	−0.02 *N* = 100	1						
11. Arts and crafts	−0.04 *N* = 94	0.03 *N* = 89	0.22* *N* = 88	0.08 *N* = 88	0.02 *N* = 95	0.07 *N* = 93	0.22* *N* = 95	0.05 *N* = 95	0.10 *N* = 95	0.25* *N* = 95	1					
12. Solitary play	0.00 *N* = 96	−0.01 *N* = 91	0.04 *N* = 90	0.09 *N* = 90	−0.15 *N* = 97	0.18 *N* = 95	0.06 *N* = 95	0.10 *N* = 96	−0.07 *N* = 97	0.08 *N* = 96	0.23* *N* = 92	1				
13. Peer play	0.04 *N* = 100	0.27* *N* = 94	0.08 *N* = 95	0.22* *N* = 95	0.18 *N* = 101	0.22* *N* = 98	0.22* *N* = 98	0.27** *N* = 98	0.24* *N* = 101	0.04 *N* = 99	−0.17 *N* = 94	−0.07 *N* = 96	1			
14. Sports and physical activities	0.27* *N* = 101	0.06 *N* = 95	0.17 *N* = 95	0.12 *N* = 95	0.11 *N* = 102	0.22* *N* = 99	0.11 *N* = 99	0.22* *N* = 99	0.44** *N* = 102	0.12 *N* = 100	0.20 *N* = 95	0.13 *N* = 97	0.14 *N* = 101	1		
15. Parental belief: academic focused	−0.01 *N* = 94	0.01 *N* = 88	0.03 *N* = 88	0.04 *N* = 88	0.32** *N* = 95	−0.06 *N* = 92	0.08 *N* = 94	−0.03 *N* = 93	0.10 *N* = 95	0.01 *N* = 94	−0.08 *N* = 91	−0.08 *N* = 91	0.14 *N* = 94	0.08 *N* = 95	1	
16. Parental belief: play support	0.22* *N* = 91	0.37** *N* = 86	0.08 *N* = 87	0.42** *N* = 87	−0.29** *N* = 92	0.44** *N* = 89	−0.07 *N* = 92	0.30** *N* = 91	0.10 *N* = 92	0.03 *N* = 91	−0.04 *N* = 89	0.22* *N* = 90	0.04 *N* = 92	0.20 *N* = 92	−0.21* *N* = 91	1

*p < 0.05; **p < 0.001.

### Hierarchical Regression Analyses

Hierarchical regression analyses were conducted to determine the contribution of children’s home activities and parental play belief variables for predicting children’s inhibitory skills, after controlling for age and SES. Accordingly, age and SES were entered first in the regression model. As shown in Table 4, this model accounted for a significant proportion of the variance (32%) on the go/no–go task performance [*F*(2, 77) = 18.49, *p* < 0.001)]. Next, based on the correlation analyses, the frequency of mealtime together with family, the frequency of having breakfast at home, the frequency of pretend play, the frequency of peer play, and parental play support were entered into the regression. The result showed that breakfast and parental play support were significant predictors in the model, while pretend play, peer play, and spending mealtime together were not. After removing pretend play, peer play, and mealtime together variables from the equation, the model was significant [*F*(4, 75) = 16.67, *p* < 0.001] and explained 47% of the variance in go/no–go task performance. Thus, breakfast at home and parental play support beliefs explained an additional 15% variance in inhibitory control. Both variables were medium-sized predictors (parental play support, β = 0.36, *p* < 0.001; frequency of breakfast, β = 0.31, *p* = 0.001).

**TABLE 4:** A hierarchical regression of inhibitory control and visual-spatial working memory using preschoolers’ home activity and parental play support variables.

	**Inhibitory control**				**Visual–spatial Working Memory**			
		
	**Step 1**		**Step 2**		**Step 1**		**Step 2**	
				
	**β**	**95% CI**	**β**	**95% CI**	**β**	**95% CI**	***B***	**95% CI**
Age	0.32	(0.02, 0.07)**	0.21	(0.00, 0.05)*	0.35	(0.02, 0.06)**	0.36	(0.02, 0.06)***
SES	0.44	(0.23, 0.58)***	0.23	(0.04, 0.39)*	0.22	(0.01, 0.31)*	0.22	(0.02, 0.31)**
Breakfast			0.31	(0.12, 0.45)**				
Play support			0.36	(0.02, 0.08)***				
Arts and crafts							0.22	(0.02, 0.24)*
F	18.49***		16.67***		8.79***		7.87***	
*R*^2^	0.32		0.47		0.18		0.23	
Adj *R*^2^	0.31		0.44		0.16		0.20	
*R*^2^-change			0.15				0.05	

*p < 0.05; **p < 0.01; ***p < 0.001; 95% CI.

Another hierarchical regression model was developed to examine the contribution of frequency of having breakfast at home and frequency of participation in arts and crafts for children’s VSWM. Breakfast experience at home and participation in art and craft were added to the model next to age and SES variables as a block. The result showed that age and SES explained 18% of the variance in Mr. Peanut VSWM [*F*(2, 79) = 8.79, *p* < 0.001]. Breakfast variable was removed from the model as its contribution was not significant. The inclusion of art and craft variable in the model improved the model’s explained variance by 5%. It was a small-sized but significant predictor (β = 0.22, *p* = 0.03). Thus, the final model explained 23% [*F*(3, 78) = 7.87, *p* < 0.001] of the total variance in Mr. Peanut VSWM task performance. The summary of the final model is presented in Table 4.

## Discussion

This study aimed to explore the relationship between preschoolers’ home activities and their EF skills, including inhibitory control, cognitive shifting, and VSWM. We also examined, for the first time as far as we know, the relationship between parental play beliefs and preschoolers’ EF skills. The findings of the study are in line with the hypothesis, suggesting a significant positive relation between parental play beliefs and their children’s EF skills, at least as far as inhibitory control is concerned.

Our results showed that the frequency with which children have breakfast at home and, interestingly, parental play support beliefs were significant predictors of preschoolers’ inhibitory control skills, after controlling for age and SES. In contrast, play frequency or parental play beliefs were not related to children’s VSWM capacity, the only significant predictor of which was the frequency of arts and crafts activities at home, after controlling for age and SES. In sum, our results support that early home experiences provide young children with opportunities that can promote their EF development (Rhoades et al., 2011). However, different activities and parental beliefs seem to have different effects on the components of EF (inhibitory control and VSWM).

Our findings that indicate a significant positive relationship among age, SES, and EF confirm the results of previous studies (Noble et al., 2005; Sarsour et al., 2011). Concerning breakfast experience, the result also supports previous findings, indicating that breakfast affects cognitive functions (Wesnes et al., 2003). One of the potential explanations for this finding could be that regular breakfast affects the nutritional value that the child receives, which could influence brain development (Datar and Nicosia, 2012). Another justification could be that having or omitting breakfast that morning could temporarily affect children’s performance of EF tasks (see Datar and Nicosia, 2012). Alternatively, this relationship might be mediated by other factors such as the effects of household chaos, which is thought to be associated with irregular and altered family meals as well as food insecurity (Fiese et al., 2016; Rosemond et al., 2019). There is also substantial evidence demonstrating that children’s chaotic home environments are linked with less optimal cognitive function (Deater-Deckard et al., 2009; Hanscombe et al., 2011).

One of the most important findings in the current study is the significant positive relationship between parental play support and preschoolers’ inhibitory control skills. This demonstrates that children with parents holding beliefs that play is an important means of development beyond amusement and valuing of play relative to more explicit academic activities at home, such as reading to a child, tend to have better inhibitory control skills. In this regard, after interpreting the results of three parental belief studies, LaForett and Mendez (2017) pointed out that parents’ play beliefs could affect the degree to which they encourage their children’s play at home, the nature of their children’s play activities, and the level of involvement in their children’s play. Consistent with this, parents with strong play support beliefs could foster opportunities for their children’s play by supplying various play resources and by actively joining them in play stimulation activities (Johnson et al., 2005). This would improve the home environment and thereby create better opportunities for children to engage in cognitively challenging activities that could contribute to their EF development. Moreover, there is also empirical evidence confirming that parental play support beliefs and academic focused beliefs were, respectively, positively and negatively related to their children’s integrative play skills (LaForett and Mendez, 2017).

A deeper level of parental involvement in their children’s play could promote better parent–child relationship, opportunities for scaffolding, attachment security, and language skill among others. There are studies that evidence a positive association of scaffolding (Hughes and Ensor, 2009; Hammond et al., 2012), attachment security (Bernier et al., 2012), and verbal ability (Landry et al., 2002) with EF skills. Bernier et al. (2010) also highlight the importance of parent–child relationship in the development of children’s self-regulatory capacities.

We speculate three reasons for the finding indicating the effects of parental play beliefs only for inhibitory control and not for VSWM. First, the result could be related to the nature of the play support belief subscale used itself. Around one-third of the items in the subscale explicitly address the importance of play in the development of social–emotional competence, which is significantly, positively linked with the development of inhibitory control (Rhoades et al., 2009). On the other hand, there is no explicitly stated item in the subscale that is related to working memory (VSWM). As a result, the subscale is expected to have more power to address inhibitory control skills than VSWM.

Second, as the ability to control behaviors and emotions in accordance with social expectations is an important part of early social competence (Rhoades et al., 2009), the parents who hold a strong play support belief would give priority to the development of these important skills. Parents’ efforts to develop such skills could directly or indirectly contribute to the development of inhibitory control. There has been also a theory and some data demonstrating that inhibitory control may facilitate the process of developing these skills (Hughes et al., 1998; Kochanska et al., 2001). As a result, parents would work on improving their children’s inhibitory control skills as a means to improve their children’s social–emotional competence. In this regard, in their study, Rhoades et al. (2009) found that inhibitory control significantly predicted social–emotional competence above and beyond other variables associated with it.

Third, many of the games that children play in their everyday lives involve the use of inhibitory control skills (e.g., Simon Says, Wesley says, traffic light, …), however, only very few of them (“memory” or arts and crafts activities) directly target VSWM. As a consequence of this, common children’s games are more likely to have a measurable facilitatory effect on inhibitory control skills than VSWM.

Another interesting result found in the current investigation is the finding depicting a significant positive association between the frequency of participation in arts and crafts activities and VSWM. It is plausible that participation in arts and crafts could contribute to a child’s ability to organize visual stimuli to create an understanding of meaningful patterns. This skill could help children to improve their VSWM. Accordingly, in the Mr. Peanut test, children with more experience in arts and crafts might be better at holding the location of the stickers in their mind than their counterparts who engage in the same activities infrequently. This situation could promote their performance in VSWM task.

The result indicating no significant relationship between parents’ academic focus beliefs and their children’s EFs might suggest that making children focus on explicit academic-related activities at home, instead of play activity, during the preschool years, could be less important for their EF development. This is also confirmed by the finding from the present study that indicates no significant relationship between the frequency of academic activity or parental academic focus beliefs at home and EF. At the same time, practical experience of preschools in Ethiopia suggests that preschoolers are engaged in far less play activity at their preschools, as most of their time is allotted for academic purpose instead (Tigistu, 2013). Therefore, parents should be aware of the importance of play for the overall development of children and promote their children’s play after preschool instead of spending much of their home time with explicit academic-related activities.

The study has some limitations. First of all, we did not manage to test our research questions on cognitive flexibility due to the small variance found on the task we used. The sample in the current study was chosen based on convenience and was not representative (for instance, we missed participants from rural areas). Children’s weight and perinatal risk factors would provide very valuable information about EFs; unfortunately, we have no data on them in this study. Moreover, as home activities were measured using individual items, the reliability of the scale was not proven. Finally, as the interpretation of the findings regarding parental beliefs and children’s activities at home (Parmar et al., 2004; Fogle and Mendez, 2006; LaForett and Mendez, 2017) needs the demographic and sociocultural contexts to be taken into account, it is questionable how the results can be generalizable to other sociocultural contexts. Therefore, subsequent work is needed to (1) test the predictors on cognitive flexibility, (2) replicate the study in different sociocultural contexts, (3) improve the validity of children’s home activities scale, and (4) determine the path by which parents’ play beliefs and preschoolers’ home activities relate to EF development. For instance, in addition to parental play belief, it would be interesting to assess the role of parent–child interactions and the nature of scaffolding during play in order to create a better understanding of the overall picture of the issue under investigation.

## Conclusion

Based on the results, we can conclude that preschoolers’ home activity and parental play support are related to children’s EF development. In general, children with parents valuing the importance of play for the overall development of children tend to have better inhibitory control skills. This is of especially high practical relevance in Ethiopia where preschoolers are being enrolled in very structured programs focusing more on literacy and far less on play (Tigistu, 2013). Besides, children who are frequently having breakfast and participate in arts and crafts activities at home tend to show better inhibitory control skills and visual–spatial working memory capacities, respectively.

## Data Availability Statement

The datasets generated for this study can be found in 4TU. ResearchData (http://doi.org/10.4121/uuid:a76e12c9-8996-4a93-be19-0ce851a335af).

## Ethics Statement

The studies involving human participants were reviewed and approved by the Eötvös Loránd University’s Research Ethics Committee (issue number: 2017/209). Written informed consent to participate in this study was provided by the participants’ legal guardian/next of kin.

## Author Contributions

BM, JF, and ZT made substantial contributions to conception, design, scale development, analysis, and interpretation of data. BM prepared the manuscript. JF and ZT read the manuscript and provided critical feedback.

## Conflict of Interest

The authors declare that the research was conducted in the absence of any commercial or financial relationships that could be construed as a potential conflict of interest.
