# Supplementary material for: The Relationship Between Parental Play Beliefs, Preschoolers’ Home Experience, and Executive Functions: An Exploratory Study in Ethiopia
Source: Front Psychol. 2020 Apr 17;11:624. doi: 10.3389/fpsyg.2020.00624 (PMC7185235; doi:10.3389/fpsyg.2020.00624)
Supplement: Supplementary file 1 [file Data_Sheet_1.PDF]

Appendix: Home Activities Scale

Direction: Please read the list of activities in the table below and rate how often they happen with your child outside of the school time. The option in the answer varies from "very rarely" [less than once a week] (1) to "very frequently" [most of the time during the day] (5).

SN	How often does the child…	Frequency				
		**Very rarely** [less than once a week] **(1)**	**Rarely** [couple of times a week] **(2)**	**Occasionally** [at least 4 times a week] **(3)**	**Frequently** [at least once a day] **(4)**	**Very frequently** [Most of the time during the day] **(5)**
1.	practice academic skills (such as studying alphabets, numbers, words…)	1	2	3	4	5
2.	spend mealtime together with parents	1	2	3	4	5
3.	have breakfast	1	2	3	4	5
4.	engage in pretend play (e.g., during playing using a stick as a spoon, pretending to drive an invisible car, using banana as a phone…)	1	2	3	4	5
5.	engage in motor play (e.g., running, climbing, jumping, throwing, balancing…)	1	2	3	4	5
6.	engage in activities such as painting, drawing, writing, cutting with scissors, scooping sand with a small shovel, grabbing mulch, ...)	1	2	3	4	5
7.	participate in art and crafts activities that involve wet textures, such as glue	1	2	3	4	5
8.	engage in solitary play	1	2	3	4	5
9.	play with peers	1	2	3	4	5
10.	do sport/physical exercise	1	2	3	4	5
